# Mechanism of decreased sensitivity of dobutamine associated left ventricular wall motion analyses for appreciating inducible ischemia in older adults

**DOI:** 10.1186/s12968-015-0131-3

**Published:** 2015-04-08

**Authors:** Sujethra Vasu, William C Little, Timothy M Morgan, Richard B Stacey, William O Ntim, Craig Hamilton, Vinay Thohan, Caroline Chiles, William Gregory Hundley

**Affiliations:** Department of Internal medicine, Section on Cardiology, Wake Forest School of Medicine, Winston Salem, North Carolina 27157 USA; Department of Internal Medicine, University of Mississippi, Jackson, Mississippi 39216 USA; Department of Biostatistical sciences, Wake Forest School of Medicine, Winston Salem North Carolina, 27157 USA; Mid Carolina Cardiology, Charlotte North Carolina, 28204 USA; Department of Biomedical Engineering, Wake Forest School of Medicine, Winston Salem, North Carolina 27157 USA; Aurora Cardiovascular Services, Milwaukee, Wisconsin 53215 USA; Department of Radiology, Wake Forest School of Medicine, Winston Salem, North Carolina 27157 USA

**Keywords:** Dobutamine, Elderly, Ischemia

## Abstract

**Background:**

Dobutamine associated left ventricular (LV) wall motion analyses exhibit reduced sensitivity for detecting inducible ischemia in individuals with increased LV wall thickness. This study was performed to better understand the mechanism of this reduced sensitivity in the elderly who often manifest increased LV wall thickness and risk factors for coronary artery disease.

**Methods:**

During dobutamine cardiovascular magnetic resonance (DCMR) stress testing, we assessed rate pressure product (RPP), aortic pulse wave velocity (PWV), LV myocardial oxygen demand (pressure volume area, PVA, mass, volumes, concentricity, and the presence of wall motion abnormalities (WMA) and first pass gadolinium enhanced perfusion defects (PDs) indicative of ischemia in 278 consecutively recruited individuals aged 69 ± 8 years with pre-existing or known risk factors for coronary artery disease. Each variable was assessed independently by personnel blinded to participant identifiers and analyses of other DCMR or hemodynamic variables.

**Results:**

Participants were 80% white, 90% hypertensive, 43% diabetic and 55% men. With dobutamine, 60% of the participants who exhibited PDs had no inducible WMA. Among these participants, myocardial oxygen demand was lower than that observed in those who had both wall motion and perfusion abnormalities suggestive of ischemia (p = 0.03). Relative to those with PDs and inducible WMAs, myocardial oxygen demand remained different in these individuals with PDs without an inducible WMA after accounting for LV afterload and contractility (p = 0.02 and 0.03 respectively), but not after accounting for either LV stress related end diastolic volume index (LV preload) or resting concentricity (p = 0.31-0.71).

**Conclusions:**

During dobutamine stress testing, elderly patients experience increased LV concentricity and declines in LV preload and myocardial oxygen demand, all of which are associated with an absence of inducible LV WMAs indicative of myocardial ischemia. These findings provide insight as to why dobutamine associated wall motion analyses exhibit reduced sensitivity for identifying inducible ischemia in elderly.

**Trial registration:**

This study was registered with Clinicaltrials.gov (NCT00542503).

## Background

The presence of inducible left ventricular wall motion abnormalities (LVWMA) observed during dobutamine stress echocardiography (DSE) and dobutamine cardiovascular magnetic resonance (DCMR) is utilized widely to identify myocardial ischemia indicative of coronary artery disease (CAD) and forecast future cardiovascular (CV) events in those unable to exercise [1-5]. Dobutamine stress wall motion analyses performed with DSE or DCMR exhibit reduced sensitivity for detecting myocardial ischemia in those with altered left ventricular (LV) geometry due to increased LV concentricity and hypertrophy, both common conditions in the elderly [6,7]. Dobutamine stress myocardial perfusion analyses performed with contrast echocardiography or cardiovascular magnetic resonance (CMR) display improved sensitivity for identifying coronary artery stenosis of intermediate severity or forecasting prognosis regardless of ventricular shape [8-10].

In this study, we sought to understand the mechanism by which myocardial perfusion defects occur without concomitant inducible LVWMA in older individuals undergoing dobutamine stress testing. To accomplish this, we performed DCMR, a procedure which allows simultaneous assessment of wall motion and perfusion, LV concentricity, preload, afterload, and contractility in older individuals who frequently exhibit altered LV remodeling.

## Methods

### Study design

The study was approved by the Institutional Review Board of Wake Forest Health Sciences, and each participant provided witnessed written informed consent. This study was registered within the United States on Clinicaltrials.gov (NCT00542503) and was performed in accordance with the National Institutes of Health grants R01HL076438 and P30AG21332. In addition, this study complied with the Declaration of Helsinki. The purpose of this initiative was to utilize advanced DCMR techniques to identify rest and stress-induced cardiac and vascular abnormalities in older individuals that are associated with CV disease. In this study, each participant underwent an interview to collect and record information pertinent to CV disease (e.g., demographics, CV risk factors, etc.) and then underwent DCMR in which rate pressure product (RPP), aortic pulse wave velocity (PWV), LV myocardial oxygen demand (pressure volume area, PVA, stroke work, SW), mass, volumes, concentricity, and the presence of WMA and first pass gadolinium enhanced perfusion defects (PDs) indicative of ischemia were measured and recorded.

### Study population

The study included participants from the rural counties of Central and Western North Carolina that exhibited a >5 years presence of risk factors (e.g., hypertension, diabetes, or CAD) for a cardiac event, but no history of myocardial infarction within 6 months of enrollment, no contraindication to intravenous dobutamine or gadolinium based contrast nor the performance of a CMR exam (such as the presence of incompatible bio-metallic implants or claustrophobia). None of the subjects had angina or symptoms of heart failure in the 12 months prior to enrollment. Recruitment of study participants was achieved through newspaper and television advertisements, or mailings to randomly selected individuals over the age of 55, but <85 years within Forsyth, Davie, and Davidson counties of Northwest North Carolina [4,5,11].

### Wall motion imaging

The DCMR protocol was accomplished according to previously published techniques [4,5] with images that were acquired on a 1.5-T (Siemens Avanto) whole-body imaging system using a phased-array cardiothoracic surface coil placed on the chest. Dobutamine was infused incrementally from low (7.5 mcg/kg/min) to peak dose (20 to 40 mcg/kg/min), along with atropine (up to 1.5 mg), to achieve 80% of the maximum predicted heart rate response (MPHRR) for age. Cine images of the left ventricle were obtained in multiple contiguous short axis slices (apex to base) and in 3 long axis views (2, 3, and 4 chamber) at rest, low and peak dose dobutamine, and then after 10 minutes of recovery. Measurements of brachial artery systolic (SBP) and diastolic blood pressure (DBP) were performed using a CMR compatible sphygmomanometer.

According to previously published techniques [11,12], LV volumes were measured from the short-axis series of cine white blood imaging sequences using a modified Simpson’s rule method [12]. LV concentricity was measured as the ratio of the LV mass to the LV end-diastolic volume as described by Chuang et al. [13]. Image acquisition parameters included a 45 msec repetition time (TR), a 1 msec echo time (TE), a 78° flip angle (FA), a 400 × 324 mm field of view (FOV), a 192 × 109 matrix, and an 8 mm thick slice with a 2 mm gap and an acceleration factor of 2 [5].

### Wall motion (WM) analysis

The LV wall motion at rest, peak dose and in recovery was assessed with a visual scoring system in which 1 = normal wall motion, 2 = hypokinesis, 3 = akinesis, and 4 = dyskinesis. An LV inducible WMA was defined as an increase in score of ≥1 (e.g., normal to hypokinetic) in 1 or more myocardial segments. Segments with an LV wall motion score of 2 or 3 at rest with no worsening of wall motion were considered negative for ischemia.

### Infarct imaging

Ten minutes after the administration of gadobenate dimeglumine (Multihance) contrast, late gadolinium enhanced (LGE) inversion recovery images with steady state free precession readout were collected in the same short axis planes used to assess LV volumes. The sequence parameters included a 6 mm thick slice with a 2 mm gap, an 800 msec TR, a 1.7 msec TE, a 40° FA, a 360 × 270 mm FOV, and a 192 × 109 matrix with an inversion time adjusted to null the myocardium. Enhanced (>3 standard deviations in mean signal intensity above background non-enhanced) regions were identified.

### Perfusion imaging

First pass perfusion imaging with gadobenate dimeglumine (0.1 mmol/kg) was performed when 80% MPHRR was achieved. Eighty percent (80%) of the maximum predicted heart rate response for age was selected for this study because [5] this heart rate response was previously shown to be accurate for identifying ischemia and forecasting cardiac prognosis [5,14]. A potential benefit of the mild decrease in peak stress heart rate (80% relative to 85%), is that it allowed for acquisition of 2 slices for assessing myocardial first pass perfusion at peak stress. These perfusion images were collected in the short axis orientation in the middle and apical segments (2 slice positions due to the rapid HR). Image parameters included an 8 mm thick slice, a 169 msec TR, a 1.1 msec TE, a FA of 12°, a FOV of 360 × 270 mm and a 192 × 108 matrix. Rest first-pass perfusion imaging was not performed.

**Figure 1:**
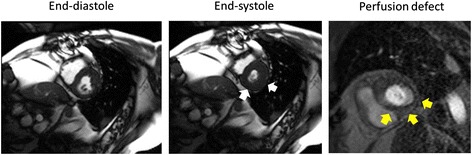
**Discordance between left ventricular wall motion abnormalities and perfusion defects indicative of inducible myocardial ischemia.** Cine white blood imaging end-diastolic (left panel) and end-systolic (middle panel) frames from slice position acquired in the middle of the left ventricle at peak dobutamine and atropine infusion administered to achieve >80% of the maximum predicted heart rate response for age. The white arrows indicate normal wall motion of the posterior and lateral wall segments. However, in the right panel, a first pass gadolinium enhanced perfusion image also acquired at peak stress is displayed. The yellow arrows indicate a hypoperfused region of the LV myocardium consistent with inducible ischemia. This participant underwent contrast coronary angiography which demonstrated a >70% stenosis of the saphenous vein graft to the right coronary artery.

For each LV myocardial segment, a two-step process was utilized to identify perfusion defects indicative of ischemia. First, regions of first-pass hypo-perfusion were measured as a percentage of the corresponding myocardial wall thickness for that LV myocardial segment in the same imaging plane (Figure 1). The radial length of the PD was expressed as a percentage of the total LV myocardial wall thickness. In addition, the duration (or number of frames) for each PD was calculated from onset of LV myocardial enhancement until complete resolution of the defect. Any PD that persisted for more than 5 frames from onset of myocardial enhancement and encompassed >25% of the thickness of the wall was further evaluated for classification as ischemic [15]. This criterion of 25% transmural involvement was used successfully by other investigators to exclude dark rim artifacts [16,17].

Further evaluation was performed to exclude PDs related to prior infarcted territories. The extent of these PDs suspected of ischemia was compared to the extent of infarction as assessed in the LGE images within the same myocardial segments. Participants with PDs that extended beyond the areas of LGE or occurred in a territory or segment that differed from the segmental territory in which the LGE was noted were classified as ischemic. Any PD that matched the area of LGE was not classified as ischemic.

### Aortic stiffness assessment using pulse wave velocity

According to previously published techniques [18], PWV was assessed using phase-contrast cardiovascular magnetic resonance (PC-CMR). Images of the proximal thoracic aorta were obtained in an axial cross-sectional plane placed at the apex of the main pulmonary artery (identified with a sagittal localizer). PC-CMR imaging parameters included an 8 mm thick slice, a 10 msec TR, a 3–5 msec TE, a 15-20°FA, 20 ms temporal resolution, a 340 to 360 mm FOV, a 256 × 192 matrix and a through plane velocity encoding of 150 cm/sec. Pulse wave velocity (PWV) was calculated by dividing the distance between the ascending and descending thoracic aorta by the transit time of the flow wave [18].

### Measures of global myocardial oxygen demand

In addition to the changes in HR, blood pressure (BP) and the RPP, we also assessed the LV stroke work (SW) and the systolic PVA, a measure indicating the global LV myocardial oxygen demand [19,20]. The systolic PVA is defined as both the mechanical LV SW and the mechanical potential energy which is expended during systole [21,22]. This is the area enclosed by the slopes of the end-systolic and the end-diastolic pressure volume relationship [22]. Invasive studies have shown an excellent correlation between the PVA and the myocardial oxygen consumption under different loading conditions and with dobutamine. The SW was defined as the mean arterial pressure * LV stroke volume. The systolic PVA was defined as: LV SW+ ½* LV end-systolic pressure * end-systolic volume. LV end systolic pressure was calculated as 0.85* brachial systolic blood pressure (SBP) [23].

### Statistical analysis

In accordance with the American Heart Association Scientific Statement [24], the 10 middle and apical LV myocardial segments were assessed for LV first pass PDs, LGE, and inducible WMA. Participants in this study were characterized into one of 3 groups including those: 1) without a PD or WMA (Group I), 2) with a PD but without a WMA (Group II), and 3) with both a PD and a WMA indicative of ischemia (Group III). The differences in demographic, hemodynamic, CMR volumetric parameters, and indices of myocardial oxygen demand between the 3 Groups were assessed by an analysis of variance test of equality (ANOVA). In Tables 1 and 2, the p-values are displayed for the overall equality of the three groups. In the Figures, the comparisons between specific groups were accomplished using pairwise comparisons. A p-value of <0.05 was considered significant for either forms of testing. The differences in the LV myocardial SW and the PVA were adjusted for preload (resting and peak dose LV end-diastolic volume index or LVEDVi), afterload (peak dose PWV), contractility (LV ejection fraction or LVEF), and LV concentricity using analysis of covariance. Multiple regression models adjusting for SBP, LVEDV baseline and LVEDV peak dose were selected and reported by stepwise regression. The sensitivity and specificity of dobutamine related wall motion abnormalities for detecting obstructive CAD was assessed with the results of the perfusion component of the DCMR protocol serving as the reference standard. Results were expressed as means ± standard error of the estimate unless stated otherwise.

**Table 1:** **Demographic data**

**Category**	**Group I No LV perfusion defect or wall motion abnormality (n = 232)**	**Group II LV perfusion defect present, no wall motion abnormality (n = 28)**	**Group III LV perfusion defect and wall motion abnormality present (n = 18)**	p-value*
Age (years)	68 ± 8	70 ± 9	69 ± 8	0.32
Men (%)	110 (47%)	14 (50%)	14 (78%)	0.046
Race/Ethnicity				0.50
Caucasian	171 (74%)	23 (82%)	16 (89%)	
African Am.	56 (24%)	4 (14%)	2 (11%)	
Hispanic	2 (1%)	1 (4%)	0 (0%)	
Asian	3 (1%)	0 (0%)	0 (0%)	
Body mass index (m/kg2)	30.4 (6.5)	30.3 (4.8)	32.1 (5.4)	0.54
Hypertension	203 (88%)	27 (96%)	16 (89%)	0.38
Coronary artery disease	57 (25%)	9 (32%)	8 (44%)	0.14
Prior MI	16 (7%)	5 (17%)	3 (18%)	0.05
Prior revascularization (PCI or CABG)	31 (13%)	9 (32%)	8 (44%)	<0.001
Diabetes	94 (41%)	11 (39%)	8 (44%)	0.94
Hypercholesterolemia	152 (70%)	22 (79%)	12 (86%)	0.30
Smoking	90 (42%)	10 (36%)	6 (43%)	0.79
Medications				
Angiotensin converting enzyme inhibitor	99 (43%)	15 (54%)	7 (39%)	0.52
Angiotensin receptor blocker	64 (30%)	5 (18%)	3 (20%)	0.30
Statin	152 (66%)	21 (75%)	14 (78%)	0.37
Beta blocker	99 (43%)	13 (46%)	11 (61%)	0.32
Calcium channel antagonist	68 (30%)	8 (29%)	9 (50%)	0.20

*Analysis of variance test of equality of three groups. Summary statistics are mean (standard error) for continuous variables and frequency (%) for categorical variables. Abbreviations: CABG, coronary artery bypass grafting; LV, left ventricular; MI, myocardial infarction; PCI, percutaneous coronary intervention.

**Table 2:** **Hemodynamics and CMR indices**

**Measure**	**Group I (n = 232)**	**Group II (n = 28)**	**Group III (n = 18)**	p-value*
**Rest**				
Heart rate (beats/min.)	65 (0.74)^**^	63.64 (2.06)	65.73 (2.81)	0.77
Systolic blood pressure (mmHg)	139.81 (1.18)	137.29 (3.29)	136.67 (4.58)	0.64
Diastolic blood pressure (mmHg)	79.53 (0.82)	75.29 (2.29)	78.80 (3.13)	0.22
Mean arterial pressure (mmHg)	99.58 (0.82)	95.95 (2.27)	98.09 (3.21)	0.30
Rate pressure product	9108 (133)	8753 (369)	8962 (504)	0.65
Left ventricular end diastolic volume index (ml/m^2^)	62.2 (1.01)	56.7 (2.81)	59.3 (3.91)	0.17
Left ventricular end systolic volume index (ml/m^2^)	22.2 (0.52)	21.3 (1.31)	22.8 (1.72)	0.72
Left ventricular stroke volume index (ml/m^2^)	39.4 (0.61)	35.5 (1.52)	36.5 (2.11)	0.03
Ejection fraction (%)	64.2 (0.51)	62.7 (1.41)	62.5 (1.82)	0.46
Cardiac index (ml/min/m^2^)	2536 (39)	2239 (109)	2380 (149)	0.03
Pulse wave velocity (m/s)	11 (0.47)	9.75 (1.28)	9.96 (1.75)	0.76
Left ventricular mass index (gm/m^2^)	66 (14)	65.9 (11.32)	71.1 (12.41)	0.35
Left ventricular concentricity	1.09 (0.02)	1.19 (0.05)	1.26 (0.07)	0.02
**Stress**				
Peak heart rate (beats/min.)	127 (1.12)	129.81 (3.16)	126.92 (4.39)	0.63
Peak systolic blood pressure (mmHg)	130 (1.75)	126.37 (4.92)	131.93 (6.84)	0.74
Peak diastolic blood pressure (mmHg)	70.79 (1.21)	68.04 (3.40)	75.14 (4.72)	0.47
Peak mean arterial pressure (mmHg)	90.57 (1.27)	87.48 (3.58)	94.07 (4.97)	0.54
Peak rate pressure product	16400 (251)	16381 (706)	16804 (1982)	0.92
Left ventricular end diastolic volume index (ml/m^2^)	51.60 (0.78)	50.99 (2.15)	54.97 (2.98)	0.52
Left ventricular end systolic volume index (ml/m^2^)	13.68 (0.33)	14.53 (0.87)	18.36 (1.24)	0.001
Left ventricular stroke volume index (SVI) (ml/m^2^)	37.85 (0.62)	36.23 (1.69)	36.64 (2.35)	0.61
Ejection fraction (%)	73.3 (0.50)	71.2 (1.42)	66.5 (1.93)	0.002
Cardiac index (l/min/m^2^)	4800 (83)	4688 (232)	4644 (316)	0.82
Pulse wave velocity (m/s)	11.51 (0.61)	12.44 (1.69)	16.27 (2.39)	0.14

*Analysis of variance test of equality of three groups. The p-values presented are an overall test that the three groups are equal. Abbreviations:, EDV, End diastolic volume; EF, Ejection fraction; PVA, Pressure volume area; PWV, Pulse wave velocity; SW, Stroke work.

## Results

Two hundred seventy-eight (278) consecutive participants were enrolled into the study; the demographic data from the participants are shown in Table 1. No differences in age, gender, BMI, prevalence of hypertension, diabetes, CAD, dyslipidemia, smoking, or medication use were noted between the 3 Groups (p = 0.05 to 0.54 for all; Table 1). Analyzing the data using pairwise comparisons, there were differences between individuals in Groups II and III versus those in Group I regarding the incidence of prior revascularization (32% and 44% vs. 13% respectively, p <0.001 for both comparisons); and prior MI (17% vs. 18% vs. 7% respectively; p = 0.05 for both comparisons). There were no differences between Groups II and III regarding the incidence or prior MI or revascularization (p = 0.98 and p = 0.29 respectively).

According to previously published criteria, 64% of the participants exhibited LV concentricity and 82% had left ventricular hypertrophy. Consistent with previously published data [25], in the population aged 70 and older, women compared to men had a greater incidence of concentric ventricles (80% vs. 42%, p < 0.001 while in those aged less than 70 there were no gender differences in the incidence of concentric ventricles (61% vs. 61%, p =0.87). However women had a higher incidence of LVH compared to men (96% vs. 62% p < 0.001).

**Figure 2:**
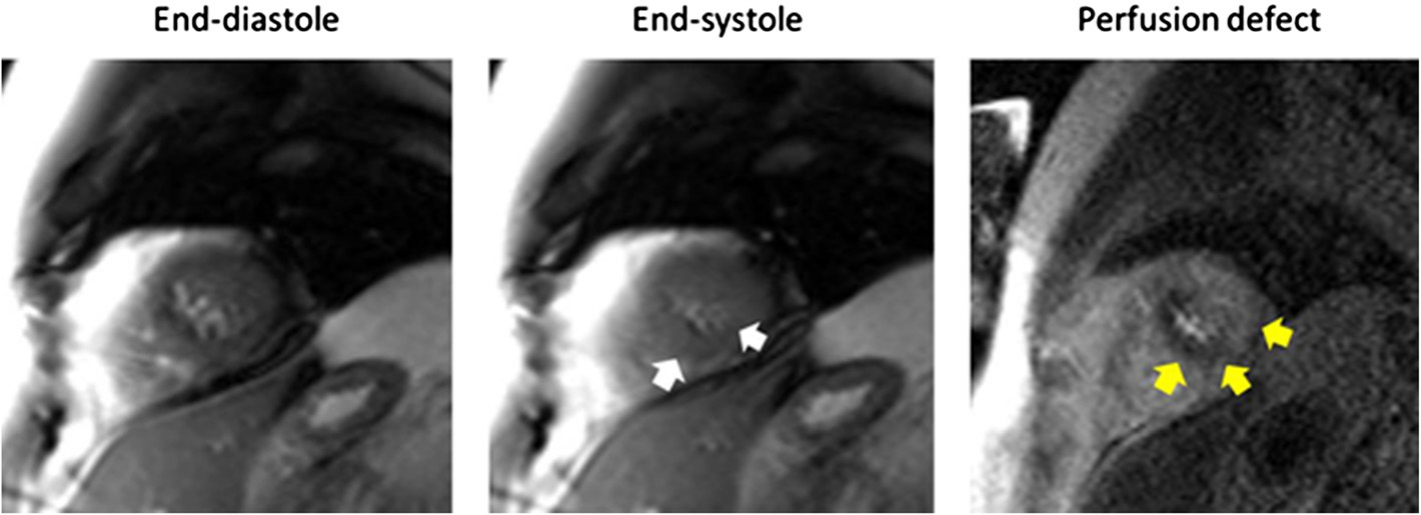
**Concordance between left ventricular wall motion abnormalities and perfusion defects indicative of inducible myocardial ischemia.** Cine white blood imaging end-diastolic (left panel) and end-systolic (middle panel) frames from slice position acquired in the apex of the left ventricle at peak dobutamine and atropine infusion administered to achieve >80% of the maximum predicted heart rate response for age. The white arrows indicate a wall motion abnormality in the inferoseptum as manifest by incomplete LV cavity obliteration at peak stress. In the right panel, a first pass gadolinium enhanced perfusion image also acquired at peak stress is displayed. The yellow arrows indicate a hypoperfused region of the LV myocardium consistent with inducible ischemia. In this case there was concordance of the wall motion and perfusion analyses both indicating inducible ischemia.

Forty-six participants exhibited a PD indicative of ischemia. Of the 46 subjects with PDs, 18 had corresponding inducible LV WMAs (Group III) [13]. The remaining 28 exhibited a PD but no WMA (Group II). No participants experienced an inducible LVWMA without a CMR perfusion defect. The presence of a DCMR inducible LVWMA exhibited a sensitivity of 39% and a specificity of 100% for the detection of a PD consistent with inducible LV myocardial ischemia. Example cases from participants with concordance and discordance between WMA and PDs are shown in Figures 1 and 2.

Of the 28 patients with perfusion defects consistent with ischemia in Group II, 6 had peri-infarct ischemia (i.e. a PD extending beyond proportion to the region of LGE), 3 had LGE in a different territory and 19 had no LGE. Of the 18 patients in Group III, 9 had periinfarct ischemia, 2 had LGE in a different territory and 7 had no LGE. Compared to the subjects in Group II, those in Group III trended toward larger trans-mural extents of their PDs (29% vs. 33%, p = 0.26), somewhat longer duration PDs (10 frames vs. 13 frames, p = 0.11), and more segments with LGE (11 vs. 18, p = 0.04). Those in Group III did exhibit a greater number of WMAs indicative of ischemia (1 vs. 4, p < 0.001 segments, respectively).

The cardiac chamber volumes and function, arterial stiffness measures, and hemodynamic parameters of the participants at rest and with stress are shown in Table 2. Systolic blood pressure (SBP), diastolic blood pressure (DBP), pulse pressure (PP), HR and the rate pressure product (RPP) were similar at rest and with stress among the 3 Groups (p = 0.22 to 0.77). Overall, with intravenous dobutamine, the HR rose 96%, the SBP decreased 6% and the RPP rose 85% for the participants in the study with no difference between the three groups (p = 0.63, 0.74, and 0.92 respectively).

At rest, those in Group I exhibited a higher LVSV index (LVSVi) and a higher cardiac index compared to those in Groups II and III (p = 0.03 for both indices). No differences between the 3 groups were noted in the LV end-systolic volume index (LVESVi) or the LVEF at rest. Those in Group III possessed the highest concentricity index (p = 0.02) compared to those in Groups I and II with no differences in the LV mass index between the three groups (p = 0.35). With dobutamine, all three groups experienced a similar decrease in the LVEDVi. As expected, individuals in Group III exhibited the lowest stress LVEF and the highest stress LVESVi values relative to those in Groups I and II (p = 0.002 and p = 0.001 respectively). PWV was not different among the groups at rest (p = 0.76) or with stress.(p = 0.14).

**Figure 3:**
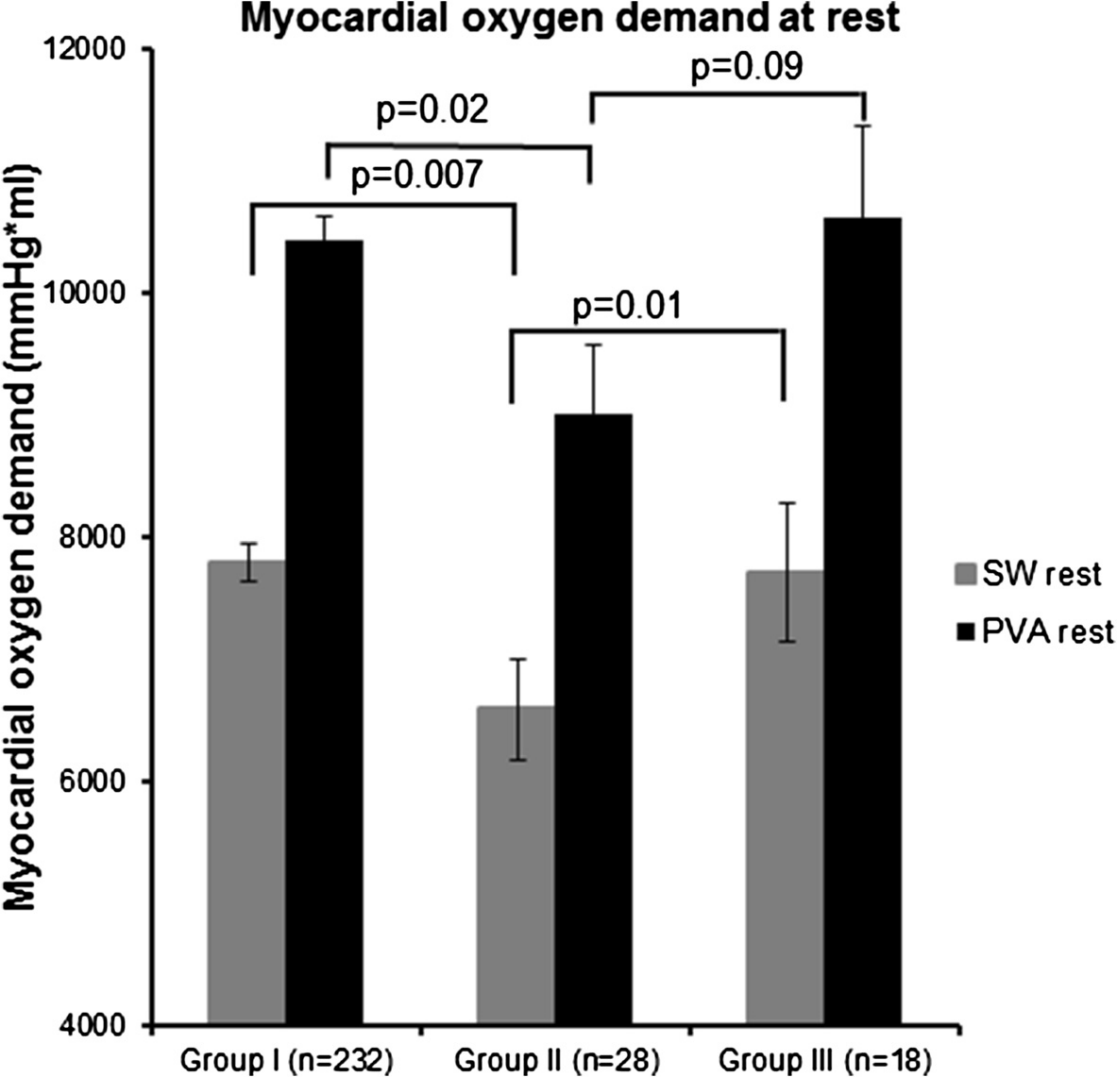
**Resting measures of myocardial oxygen demand between the three groups.** The mean ± the standard error of the myocardial oxygen demand (Y-axis) and the number of participants in each of the 3 study groups (X-axis) are shown. The myocardial stroke work is denoted by the gray bars, and the pressure volume area is denoted by the black bars. At rest, Group II participants (wall motion negative, perfusion positive) have lower myocardial oxygen demand as assessed by myocardial stroke work (SW) than those in III (wall motion and perfusion positive) or Group I. Also, PVA is reduced in Groups II relative to Group I and trends toward a difference with Group III.

**Figure 4:**
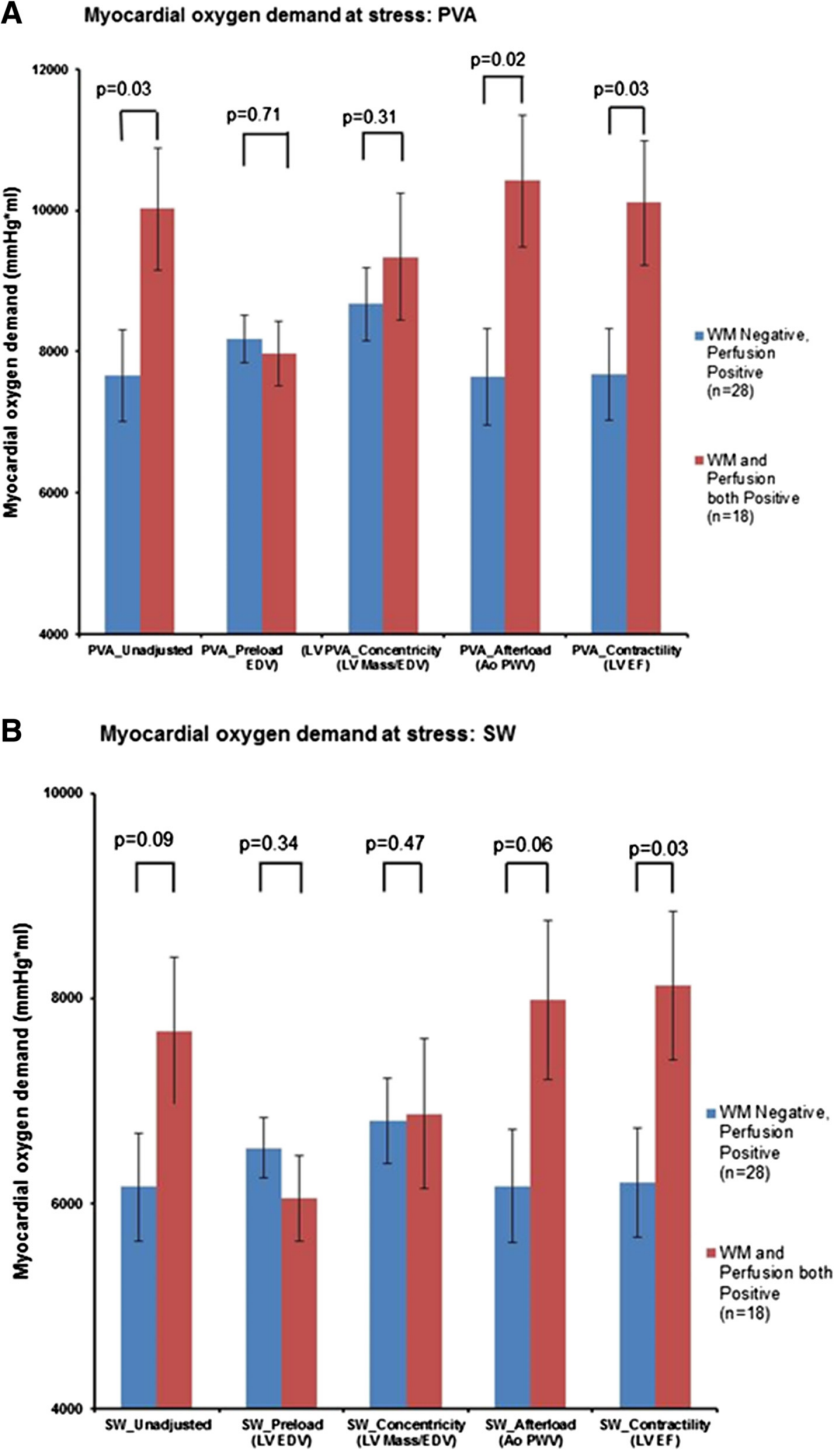
**Adjusted and unadjusted measures of myocardial oxygen demand. A** shows the unadjusted differences in myocardial oxygen demand with stress, specifically the PVA, between Groups II and II. The differences in the unadjusted PVA between Groups II and III are attenuated when adjusted for LV preload (LV End-diastolic volume (LVEDV)) and LV concentricity, but persist when adjusted for LV afterload (Aortic pulse wave velocity (PWV) and LV contractility (LV ejection fraction (LVEF). **B** demonstrates the differences in Stroke work between Groups II and III. Similar to PVA, LV Preload and Concentricity determine the differences in the Stroke Work between Groups II and III, but not the LV afterload or contractility. **A** and **B** show the attenuation of these differences when adjusted for LV preload and concentricity respectively. This suggests that LV preload and concentricity are the main factors influencing myocardial oxygen demand.

The indices of myocardial oxygen demand at rest are shown in Figure 3 and at stress in Figure 4. At rest as shown in Figure 3, the left ventricular pressure volume area (PVA) and stroke work (SW) of individuals in Group II were lower than those in Group I (9,016 ± 559 vs. 10,439 ± 201 mmHg*ml, p = 0.02 and 6,594 ± 417 vs. 7,794 ± 150 mmHg*ml, p = 0.007 respectively) and lower than those in Group III (9016 ± 559 vs. 10,618 ± 768 mmHg*ml, p = 0.09 and 6,594 ± 417 vs. 7708 ± 569 mmHg*ml, p = 0.01 respectively). However there were no differences in PVA and SW between Groups I and II with dobutamine (7,660 ± 645 vs. 8,409 ± 227 mmHg*ml, p = 0.27 and 6,160 ± 524 vs. 6,877 ± 187 mmHg*ml, p = 0.25 respectively). In contrast, during stress, the PVA and SW in Group II were lower when compared to Group III (7660 ± 645 vs. 10,023 ± 862 mmHg*ml, p = 0.03, and 6,160 ± 524 vs. 7682 ± 715 mmHg*ml, p = 0.09), respectively.

To determine the factors influencing this lower myocardial oxygen demand between groups II and III, we adjusted the left ventricular PVA and SW for differences in baseline and peak stress LVEDVi (preload), peak stress PWV (afterload), the resting LVEF (contractility), and the LV concentricity as shown in Figure 4A and B respectively. As shown in Figure 4A, the lower LVPVA in individuals in Group II relative to Group III persisted after adjusting for contractility (7,684 ± 649 vs. 10,109 ± 883 mmHg*ml, p = 0.03), and afterload (7,639 ± 682 vs. 10,421 ± 930 mmHg*ml, p = 0.02). However, the differences in the PVA after dobutamine between individuals in Groups II and III were eliminated after accounting for stress LVEDVi (p = 0.71, Figure 4A), and LV concentricity (p = 0.31; Figure 4A). Similarly, the lower LVSW in individuals in Group II relative to Group III persisted after adjusting for contractility (6,206 ± 529 vs. 8123 ± 720 mmHg*ml, p = 0.03; Figure 4B), and nearly persisted after accounting for LV afterload (6,170 ± 556 vs. 7,982 ± 776 mmHg*ml, p = 0.06; Figure 4B), but as shown in Figure 4B, were eliminated when adjusted for stress preload, LVEDVi (p = 0.34) and LV concentricity (p = 0.47). This suggests that the lower LVSW and the PVA seen in individuals in Group II are related to the LV preload and LV concentricity. Similar results between Groups II and III were noted when the PVA and SW were respectively adjusted for baseline LVEDVI (8,207 ± 555 vs. 9,534 ± 740 mmHg*ml, p = 0.15, and 6,586 ± 459 vs. 7,294 ± 624 mmHg*ml, p = 0.36.

## Discussion and conclusions

There are three important findings related to this study. First, in older men and women receiving a pharmacologic dobutamine stress test, myocardial oxygen demand often decreases during receipt of intravenous dobutamine despite achieving 80% of the MPHRR for age (Figure 4A). Second, this reduction in myocardial oxygen demand is related to altered LV geometry and reductions in LV preload that are manifested by a decrease in LVEDV upon receipt of intravenous dobutamine (Figure 4A and B). Third, dobutamine related reductions in myocardial oxygen demand observed in older men and women may confound the identification of LV inducible wall motion abnormalities indicative of ischemia (Figures 1, 2, 3 and 4). In this study of elderly subjects at risk for inducible ischemia, of those exhibiting PDs suggestive of ischemia, 60% of individuals did not demonstrate a corresponding inducible LV wall motion abnormality.

As shown in Figures 1 and 2 and Table 1, we identified three groups of individuals within the study population: those without (Group I) or with (Group III) inducible PDs and WMAs, and those with an inducible PD but without an inducible LV WMA indicative of ischemia (Group II). Similar observations in which PDs occur in the absence of dobutamine induced WMA have been reported in patients with resting LV hypertrophy [26]. In those with LV hypertrophy, the presence of PDs has an increased sensitivity when compared to inducible LV WMA for detection of coronary arterial luminal narrowings of >70% [26]. This is similar to observations made in studies of patients with altered LV geometry using both echocardiography and CMR [6,7]. The addition of myocardial perfusion has previously exhibited diagnostic and prognostic value with dobutamine stress testing, regardless of whether echocardiography [7,8] or CMR imaging [27,28] was used for diagnosis.

To further understand why PDs occurred in the absence of WMA, we examined multiple hemodynamic variables throughout the stress testing procedure. As shown in Table 2, HR increased while SBP decreased in all 3 participant groups (Table 2). This decrease in SBP contrasts with observations in prior studies of younger individuals in which a hypertensive response was uniformly noted after receipt of dobutamine [29]. As a result, the RPPs of the participants in this study ranged from 16,000 to 17,000 which are somewhat lower values than reported in previous studies of dobutamine stress testing, even after accounting for participant age [26,30]. This observation may be related to the fact that elderly individuals often exhibit increased ventricular and arterial stiffness. As a result, their LV end-systolic pressure volume relationship curve is steeper which can potentially lead to greater reductions in SBP after the administration of systemic vasodilators when compared to younger individuals with more compliant arterial systems [30].

PWV, a measure of arterial stiffening, was mildly elevated at rest across the 3 participant Groups. With stress, PWV remained similar or increased in all 3 Groups with the largest increase occurring in Group III participants. These data indicate that overall, aortic stiffness was elevated in older subjects and remained elevated after receipt of dobutamine. Given the absence of a concomitant increase in SBP in Group III, one possible explanation of the high PWV is that this group did not experience a vasodilator response with dobutamine stress. We have shown that elderly subjects with diabetes and impaired fasting glucose do not experience a normal decrease in afterload with dobutamine and instead experience an increase in arterial stiffness as evidenced by a decrease in aortic distensibility [31].

As shown in Figure 4A, myocardial oxygen demand as assessed by PVA was diminished at peak stress in those individuals with PDs but no WMAs (Group II) relative to those individuals in Group III (p = 0.03). We sought to determine which aspect (LV preload, LV afterload, LV contractility, or LV concentricity) of myocardial oxygen demand accounted for these differences by adjusting for each variable in our linear regression models. As shown in Figures 4A and B, after accounting LV preload and concentricity, the differences in myocardial oxygen demand between Groups II and II were eliminated. This implies that both of these variables were integral for establishing the difference in myocardial oxygen demand noted between these two Groups.

Since LV wall motion assessments of ischemia are driven by a supply–demand mismatch, a lower oxygen demand might not be adequate to provoke a WMA and could render the study insensitive for the detection of ischemia. The observation of a lower myocardial oxygen demand in those with increased LV concentricity, leading to hyperdynamic (and, consequently, and absence of WMA even though ischemia may be present during intravenous dobutamine) has been demonstrated by Mirelis, et al. [32]. These investigators identified fewer DCMR WMA in individuals with increased LV concentricity.

Consistent with findings from other larger population based studies such as the Multi-Ethnic Study of Atherosclerosis, the elderly individuals in our group exhibited a smaller LVEDV and increased LV concentricity [28]. In addition, as reported in other studies, the LVEDVi decreased during dobutamine [32]. However, the magnitude of the decrease in LVEDVi was higher in this study relative to observations in prior studies of middle aged individuals undergoing dobutamine stress [11]. In the presence of concentric LV remodeling or hypertrophy, decreases in LV preload can reduce the LV cavity size and render it more difficult to visualize WMAs even when myocardial shortening is abnormal [29].

The results of this study have important implications for the interpretation of dobutamine wall motion stress testing in elderly individuals. As shown in this study, up to 61% of subjects who had ischemia identified by PDs did not experience an inducible WMA. In addition, the presence of these PDs was not associated with failure to achieve target HR during testing, or differences in the SBP response (which parenthetically decreased during testing) among all individuals receiving testing. Therefore, inducible ischemia (as defined by an inducible PD) may in fact be present during dobutamine stress in the elderly even though LV WMAs are absent. Other studies have found similar results regarding the lower sensitivity of LVWMA measures in assessing the risks for CAD [6,28].

Our study does exhibit some limitations. First, we did not perform contrast coronary angiography on the participants. Recently, however, the diagnostic utility and accuracy of CMR perfusion abnormalities have been established in multicenter and multivendor trials [33,34,35]. Second, the majority of our subjects were Caucasian. Insufficient numbers of individuals from other ethnicities were present to examine the effects of race or ethnicity. Third, our study population exhibited pre-existing CAD and multiple risk factors for CAD with either no or stable patterns of angina. More research is required to determine results in subjects with new onset, or unstable patterns of angina.

In conclusion, despite achieving target HR, a subset of elderly patients with risk factors for CAD who undergo dobutamine stress experience perfusion abnormalities indicative of ischemia without a concomitant LV wall motion abnormality. Mechanistically, this occurs in part due to a lower myocardial oxygen demand that appears related to reductions in LV preload and increases in LV concentricity.
